# Common double-lumen tube selection methods overestimate adequate tube sizes in individual patients – a 3D reconstruction study

**DOI:** 10.1186/s12871-024-02605-7

**Published:** 2024-07-01

**Authors:** Lorenz L. Mihatsch, Sandra Weiland, Thomas Helmberger, Patrick Friederich

**Affiliations:** 1 Technical University of Munich, Germany, TUM School of Medicine and Health, Munich, Germany; 2 Department of Anaesthesiology, Critical Care Medicine and Pain Therapy, Munich Clinic Bogenhausen, Academic Teaching Hospital of the Technical University of Munich, Munich, Germany; 3 Institute for Medical Information Processing, Biometry, and Epidemiology, Ludwig- Maximilians-Universität München, Munich, Germany; 4 Department of Radiology, Neuroradiology and Minimally Invasive Therapy, Munich Clinic Bogenhausen, Academic Teaching Hospital of the Technical University of Munich, Munich, Germany

**Keywords:** Double-lumen tube, Left bronchus, Prediction, Bronchial damage

## Abstract

**Background:**

Appropriate selection of double-lumen tube sizes for one-lung ventilation is crucial to prevent airway damage. Current selection methods rely on demographic factors or 2D radiography. Prediction of left bronchial diameter is indispensable for choosing the adequate tube size. This prospective observational study investigates if current selection methods sufficiently predict individuals’ left bronchial diameters for DLT selection compared to the 3D reconstruction.

**Methods:**

100 patients necessitating thoracic surgery with one-lung ventilation and left-sided double-lumen tubes, ≥ 18 years of age, and a set of chest X-rays and 2D thorax CT scans for 3D reconstruction of the left main bronchus were included between 07/2021 and 06/2023. The cross-validated prediction error and the width of the 95%-prediction intervals of the 3D left main bronchial diameter utilizing linear prediction models were based on current selection methods.

**Results:**

The mean bronchial diameter in 3D reconstruction was 13.6 ± 2.1 mm. The ranges of the 95%-prediction intervals for the bronchial diameter were 6.4 mm for demographic variables, 8.3 mm for the tracheal diameter from the X-ray, and 5.9 mm for bronchial diameter from the 2D-CT scans. Current methods violated the suggested ‘≥1 mm’ safety criterion in up to 7% (men) and 42% (women). Particularly, 2D radiography overestimated women’s left bronchial diameter. Current methods even allowed the selection of double-lumen tubes with bronchial tube sections greater than the bronchial diameter in women.

**Conclusions:**

Neither demographic nor 2D-radiographic methods sufficiently account for the variability of the bronchial diameter. Wide 95%-prediction intervals for the bronchial diameter hamper accurate individual double-lumen tube selection. This increases women’s risk of bronchial damage, particularly if they have other predisposing factors. These patients may benefit from 3D reconstruction of the left main bronchus.

**Trial registration:**

Not applicable.

**Supplementary Information:**

The online version contains supplementary material available at 10.1186/s12871-024-02605-7.

## Background

Double-lumen tubes (DLTs) are the preferred device for one-lung ventilation [1–3]. Selecting adequate left DLT sizes remains a matter of debate [4–9]. Airway ruptures are the most feared complication in DLT placement, occurring primarily in the distal trachea or the main bronchus in 52.4% and 37.4% of the reported incidences, respectively [10, 11]. Most frequent DLT-related factors contributing to airway ruptures include over-sized tubes and cuff-overdistention, i.e., the use of under-sized tubes or factors predisposing to bronchial damage [10]. Current methods in use for DLT size selection rely either on patient’s demographics, i.e., sex and height, as suggested by Slinger et al., on 2D radiography, i.e., tracheal diameter measured in chest X-rays, as suggested by Brodsky et al., or on left bronchial diameters measured in 2D thorax CT scans (2D-CT), as suggested by Hannallah et al. [5, 12, 13]. Though no current selection methods prevailed so far, it seems a consensus that the left bronchus is one of the critical anatomical structures to approximate [8].

The rational is to select a DLT small enough to prevent bronchial damage, with bronchial rupture as an extremely rare but most severe complication, while choosing a tube large enough to minimize airway resistance and avoid cuff overblocking, minimizing the risk of mucosal damage [14–17]. Thus, it has been recommended to select the largest DLT that adheres to the criterion of having a ≥ 1 mm difference in diameter between the patient’s bronchial diameter and the DLT’s outer bronchial diameter [18]. Hence, relying on a criterion such as the ≥ 1 mm criterion requires accurate prediction up to the millimetre. As women and Asians have smaller bronchial diameters, the tolerance margin for incorrect predictions is smaller, making them more prone to erroneous predictions [19]. Recent evidence indicates bronchus ruptures are about twice as common in women than in men [10]. Some authors even advocate modified selection criteria for Asian populations with DLTs < 35 French (Fr) to be used [17]. As small DLTs pose a greater risk of dislocation, poorer suction conditions and may make bronchoscopy impossible, a general recommendation for small DLTs < 35 Fr may not be advisable [20, 21]. Therefore, it remains an open question as to who could benefit from smaller < 35 Fr DLT in light of alternative procedures [22, 23].

It is essential to note that the size of a DLT, measured in French, only reflects the tracheal section, and currently, no industrial norm for the bronchial section of the tube exists [24, 25]. It is further known that the DLTs’ bronchial diameter can vary considerably across different manufacturers and even within single manufacturers [24, 26–28], making it unclear whether selection methods reliant on 2D radiography and DLTs of one manufacturer apply to other manufacturers [12, 13]. Slinger et al. does not even refer to any particular manufacturer [5].

This prospective study investigated whether 3D reconstructions from 2D-CT scans can accurately determine patients’ left bronchial diameters. Compared with 2D-CT scans, 3D reconstruction is less prone to cutting artifacts [29–31]. The objective of this study was:

1. Determining the prediction error of demographic and 2D-radiographic methods for the patient’s bronchial diameter, determined by 3D reconstruction.
2. To evaluate current selection methods about the ≥ 1 mm criterion based on 3D-reconstructed bronchial diameters.
3. To identify a subpopulation that may benefit from a 3D reconstruction of the left main bronchus for DLT selection.

## Methods

This prospective observational study considered patients undergoing thoracic surgery necessitating one-lung ventilation at München Klinik Bogenhausen (Munich, Germany) from 07/2021 to 12/2021.

### Ethics

All patients gave written informed consent before inclusion in the study in agreement with the Bavarian Hospital Law (BayKrG § 27). Ethical approval was waived by the ethical committee of the Bayerische Landesärztekammer, Munich, Germany (Reference number: 2021 − 1165). The study adheres to the Declaration of Helsinki and its later amendments.

### Inclusion criteria

The necessity for thoracic surgery requiring one-lung ventilation. Other inclusion criteria were: (1) use of left-sided DLTs, (2) ≥ 18 years of age, and (3) complete set of routine chest X-ray and CT scans of the thorax.

### Data generation

Demographic variables were assessed at study inclusion. Outer diameters of the bronchial tube sections, proximal of the bronchial cuff, were retrieved from the literature for manufacturers Rüsch (Rüsch Bronchopart^®^, Teleflex, Athlone, Ireland), Sheridan (Hudson RCI^®^ Sheridan^®^ SHER-I-BRONCH^®^ endobronchial tube, Teleflex, Athlone, Ireland), Portex (Portex Blue Line^®^ endobronchial tube, Smiths Medical, Hythe, UK), VIVASIGHT (VIVASIGHT-DL^®^, ETView Medical, Misgav, Israel) and Well Lead (Well Lead^®^ endobronchial tube, Well Lead Medical, Panyu, China) [24]. For Mallinckrodt (Mallinckrodt^®^ endobronchial tube, Broncho-Cath, Dublin, Ireland), only the tubes’ outer bronchial diameters distal of the bronchial cuff were available for all sizes [32]. Corresponding diameters are shown in the Supplementary Table S1. The outer bronchial diameters of EPSA (EPSA^®^ double-lumen bronchial tube, Electroplast S.A., Montevideo, Uruguay) DLTs were measured from 141 tubes (30 of size 35 Fr, 40 of size 37 Fr, 53 of size 39 Fr and 18 of size 41 Fr) using an analogue sliding calliper with a measurement error of ± 0.05 mm (Hoffmann Group, Munich, Germany) in triplicates. Acknowledging the oval shape, the largest and smallest diameter proximal to the bronchial cuff were determined and averaged for each DLT. The resulting diameters are presented in the Supplementary Table S2.

### Radiographic measurements

The tracheal diameter from anterior-posterior chest X-rays and 2D-CT scans were measured interclavicularly with the cursor (Supplementary Fig. S1A). All chest X-rays were performed using a standard quality approved technique: upright patient position, anteriorly positioned towards the detector, with a distance of the X-ray source (focus) to the detector of 180 cm. For our measurements, we assumed that the trachea is anatomically located in a rather central thorax position, resulting in an estimated magnification factor of 1.05 to 1.20. The left bronchial diameter was measured in 2D-CT scans 10 mm distal of the carina from the coronary axis (Supplementary Fig. S1B). Further, the 2D-CT scans were used to create 3D reconstructions of the airways using 3D reconstruction software (IntelliSpace Portal 12, Philips, Amsterdam, Netherlands). From the 3D reconstruction, the perfect cross-section of the airways was created automatically, and the average diameter of the airway was calculated by the software for each slice. The bronchial diameter in 3D reconstruction was measured 10 mm distal of the carina, and the tracheal diameter respectively 60 to 70 mm proximal to the carina (Supplementary Fig. S1C, D).

### Statistical analysis

All statistical analysis was calculated in R version 4.2.1 “Funny Looking Kid” (The R Foundation for Statistical Computing, Vienna, Austria). A data analysis and statistical plan were written after the data were accessed. Mean and SD are given as mean ± SD. Various statistical tests (χ^2^-test, Fisher Exact-test, Wilcoxon rank sum-test, and t-test) were used as appropriate to compare men and women. Linear regression models were used to predict bronchial and tracheal diameters, including an interaction effect of sex and body height as indicated. Leave-one-out cross-validation was used to quantify the prediction errors, i.e., the difference between the measured bronchial or tracheal diameter from 3D reconstruction and the predicted diameters from models, reliant on demographic variables of 2D radiography. Bland-Altman plots were utilized to compare radiographic methods. Type I error of statistical tests was set to a maximum of α = 0.05. Levels of significance are indicated by *P* < 0.1., *P* < 0.05*, *P* < 0.01**, and *P* < 0.001***.

#### Sample size

To explain ≥ 20% (R^2^ ≥ 0.20) of the observed variability of the bronchial diameters in 3D reconstruction using a linear regression model with ≤ 2 independent predictors demanding power of at least 80% and a level of significance of 5%, a sample size of at least 42 patients is required. Since no prior information on the error rate of DLT selection was available, we deliberately set the sample size to *N* = 100.

#### Prediction intervals

Supplementary Fig. S2 emphasizes the difference between confidence and prediction intervals. It includes three subplots (A), (B), and (C) with equal slopes. All slopes are significantly different from zero, indicating that the predictor on the x-axis significantly affects the variable to predict on the y-axis. However, the predictor in subplot (C) explains more variance compared to subplots (A) and (B). As a result, the prediction in subplot (C) is much more accurate than in subplots (A) and (B). This emphasizes the importance of considering both confidence and predictability. Since prediction intervals are insensitive to linear transformations of the independent variables, the span of the prediction intervals, i.e., the prediction quality, does not depend on the magnification factor used for the tracheal diameter in the chest X-ray [33].

## Results

Of 189 patients necessitating one-lung ventilation in our study period, 52 were not included due to missing study personnel, 24 were re-operated, 12 did not meet the inclusion criteria, and one did not consent. Thus, 100 patients were included. Patient characteristics and radiographic measurements are shown in Table 1. All variables except age were significantly different between men and women.

**Table 1:** Patients’ characteristics and measured tracheal and left bronchial diameters

Variable	Total	Men	Woman	*P* – Value
	*n* = 100	*n* = 57	*n* = 43	
Age [years]	65.6 ± 13.5	67.1 ± 12.7	63.6 ± 14.4	0.210
Body height [m]	1.72 ± 0.11	1.78 ± 0.08	1.63 ± 0.07	< 0.001***
Weight [kg]	75.7 ± 17.3	82.7 ± 14.9	66.3 ± 15.9	< 0.001***
BMI [kg/m^2^]	25.6 ± 5.0	26.1 ± 4.7	24.9 ± 5.4	0.036*
RTx – tracheal ∅ [mm]	19.2 ± 3.3	20.3 ± 3.7	17.9 ± 2.1	< 0.001***
2D-CTx – left bronchial ∅ [mm]	14.7 ± 2.5	16.0 ± 2.1	12.9 ± 1.7	< 0.001***
3D-CTx – left bronchial ∅ [mm]	13.6 ± 2.1	14.8 ± 1.8	11.9 ± 1.2	< 0.001***
3D-CTx – tracheal ∅ [mm]	18.3 ± 2.9	19.9 ± 2.6	16.3 ± 1.4	< 0.001***

RTx: chest X-ray; 2D-CTx: 2D thorax CT scan; 3D-CTx: 3D reconstruction from 2D thorax CT scan

t-Test and Wilcoxon rank sum tests were used when appropriate

Data is given as mean ± SD

Our first objective was to analyse the predictability of patients’ bronchial diameters, measured in 3D reconstruction, by demographic variables and 2D-radiographic measurements. Significant differences were observed in bronchial diameter measurements (2D-CT vs. 3D reconstruction, *P* < 0.001***) and tracheal diameter measurements (chest X-ray vs. 3D reconstruction, *P* = 0.040*). 2D-CT scans overestimated bronchial diameters, and chest X-rays overestimated tracheal diameters compared to 3D reconstruction. Notably, 2D-CT scans showed a significant negative bias for larger bronchial diameters (*P* = 0.017*, Supplementary Fig. S3). Prediction criteria for the following three models are summarised in Table 2.

**Table 2:** Prediction criteria of the three linear models for the bronchial diameter

Model	*R*-squared^a^	Mean PE^a^	Median PE^a^	Max Span of 95%-Prediction Interval
Demographic	0.49	1.5 mm	1.2 mm	6.4 mm
RTx	0.46	1.5 mm	1.2 mm	8.2 mm
2D-CTx	0.52	1.4 mm	1.1 mm	5.9 mm

Demographic: Sex and body height; RTx: bronchial diameter measured in chest X-ray; and 2D-CTx: bronchial diameter measured in 2D thorax CT scan used as predictor variables

PE: Prediction error; ^**a**^: cross-validated parameter

### Prediction of bronchial diameter by sex and body height

**Fig. 1:**
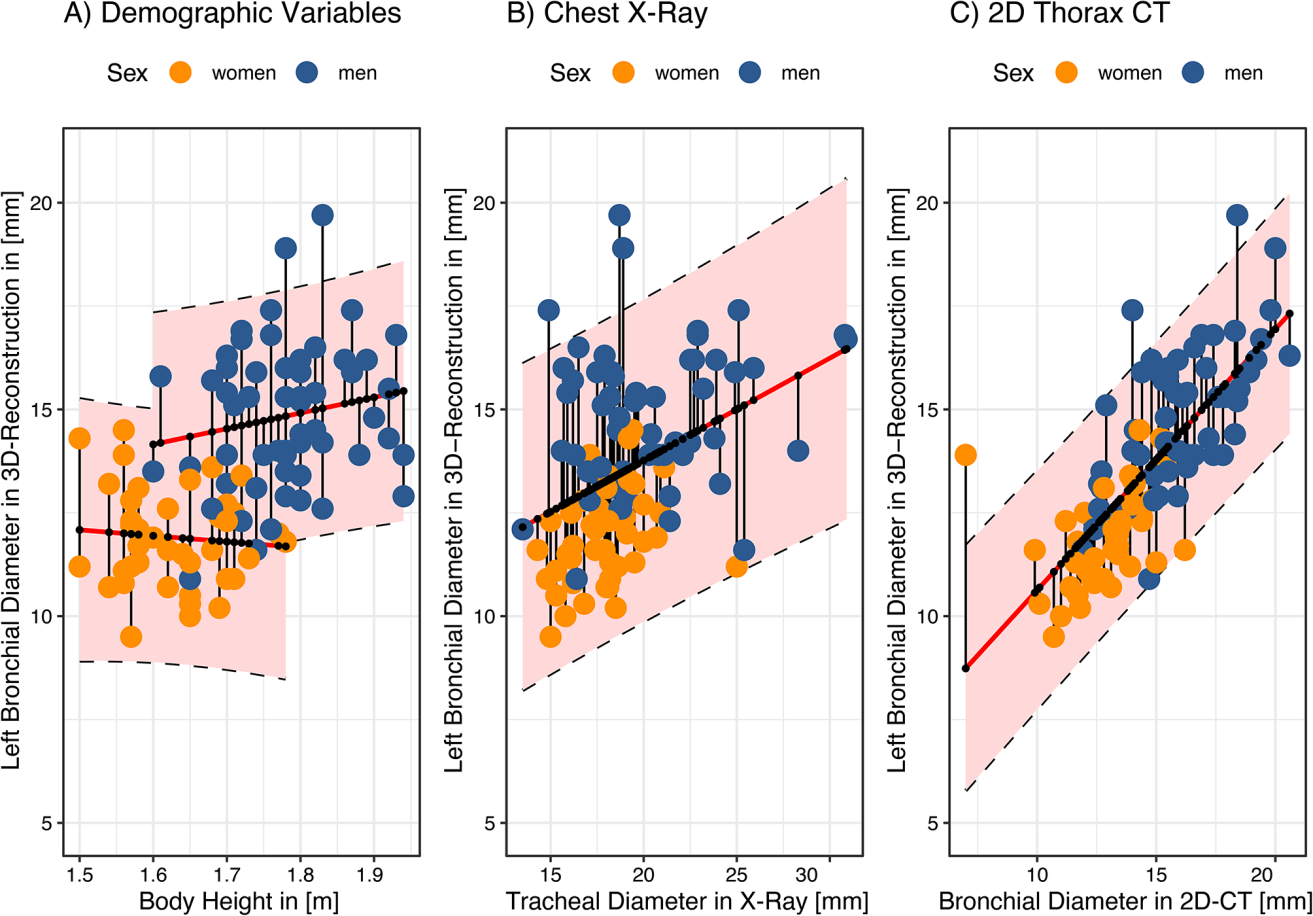
Prediction of left bronchial diameter from 3D reconstruction depending on (**A**) demographic variables, (**B**) tracheal diameter in the chest X-ray and (**C**) left bronchial diameter in 2D-CTs by linear models. Measured bronchial diameters are given as coloured dots depending on sex. Red lines show the predicted bronchial diameters and the 95%-prediction intervals as shaded areas. Vertical black line segments indicate the difference between measured and predicted bronchial diameters (individual prediction errors)

The linear prediction model for patients’ bronchial diameters by demographic variables is shown in Fig. 1A. On average, the difference between measured and predicted bronchial diameter (mean prediction error) is 1.5 mm. The 95%-prediction intervals span a range of up to 6.4 mm. The analogue model for the tracheal diameter prediction is shown in the Supplementary Fig. S4A.

### Prediction of bronchial diameter by chest X-ray

The linear prediction model for patients’ bronchial diameters by the tracheal diameter from a chest X-ray is shown in Fig. 1B. The bronchial diameter is systematically overestimated in women. For men, the bronchial diameter is systematically underestimated. The mean prediction error is 1.5 mm. The 95%-prediction interval spans a range of up to 8.2 mm. The analogue model for the tracheal diameter prediction is shown in the Supplementary Fig. 4B.

### Prediction of bronchial diameter by 2D-CT

**Fig. 2:**
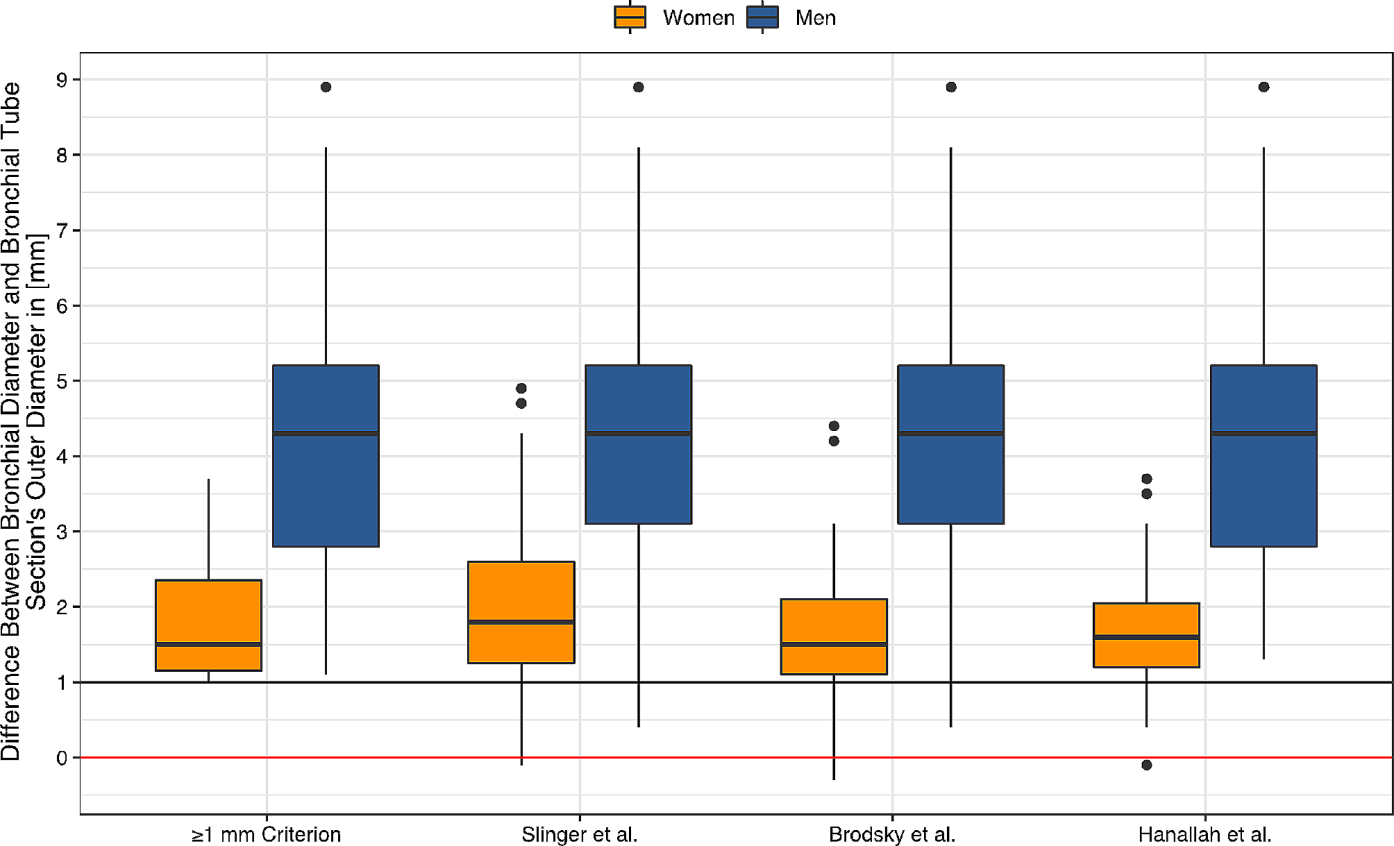
Difference between patients’ bronchial diameters and tubes’ outer bronchial diameters for chosen tubes depending on the selection method and sex. Observations below the horizontal 1 mm line (black line) do not meet the ≥ 1 mm criterion; below the red line indicate individuals whose diameter of selected tubes’ outer bronchial diameter is larger than the patients’ bronchial diameters

The linear prediction model for 3D-reconstructed bronchial diameter by the bronchial diameters measured in 2D-CT is shown in Fig. 2C. Again, the bronchial diameters of women are over-, and of men underestimated. The mean prediction error is 1.4 mm. With 5.7 mm, the span of the 95%-prediction interval is the lowest among all three models (Table 2). The analogue model for the 3D-reconstructed tracheal diameter predicted from the tracheal diameter measured in 2D-CTs is shown in Supplementary Fig. S4C.

### Comparison of selection methods

Table 3 shows the proposed tube sizes resulting from currently used selection methods: the demographic method based on sex and body height (Slinger et al.) [5], the 2D-radiographic selection methods based on the tracheal diameter on a chest X-ray (Brodsky et al.) [12], and the bronchial diameter were using a 2D-CT (Hannallah et al.) [13]. Since the tube’s outer bronchial diameter heavily depends on the manufacturer (Supplementary Tables S1 and S2), the ≥ 1 mm criterion was exemplarily applied to Mallinckrodt tubes in Table 3.

**Table 3:** Double-lumen tube sizes were selected depending on the selection method. The ≥ 1 mm criterion was exemplarily applied to Mallinckrodt double-lumen tubes

Fr	≥ 1 mm criteria			Slinger et al. [5].			Brodsky et al. [12].			Hannallah et al. [13].		
	women	men	total	women	men	total	women	men	total	women	men	total
**32**	5 12%	0 0%	5	0 0%	0 0%	0	0 0%	0 0%	0	0 0%	0 0%	0
**35**	5 12%	1 2%	6	17 40%	0 0%	17	5 12%	0 0%	5	10 23%	1 2%	11
**37**	6 14%	0 0%	6	26 60%	0 0%	26	10 23%	2 4%	12	13 30%	1 2%	14
**39**	5 12%	1 2%	6	0 0%	6 11%	6	22 51%	10 18%	32	12 28%	7 12%	19
**41**	22 51%	55 96%	77	0 0%	51 89%	51	6 14%	45 79%	51	8 19%	48 84%	56

Data is given as n (%)

Selected tube sizes significantly differed between the methods (*P* < 0.001***, χ^2^-test) and between the sexes (*P* < 0.001*** for all four methods, Fisher Exact-tests). The ≥ 1 mm criterion selected 41 Fr sizes in 96% of all men and 32 Fr sizes in 12% of all women. Other methods selected no 32 Fr tubes and 79 to 89% 41 Fr tubes for men. The application of the ≥ 1 mm criterion to other manufacturers’ DLTs is presented in the Supplementary Table S3.

**Table 4:** The difference between patients’ bronchial diameter and the selected tubes’ outer bronchial diameter depends on the selection method for Mallinckrodt double-lumen tubes

Difference	≥ 1 mm criteria		Slinger et al. [5].		Brodsky et al. [12].		Hannallah et al. [13].	
	women	men	women	men	women	men	women	men
**< 0 mm**	0 0%	0 0%	2 5%	0 0%	6 14%	0 0%	3 7%	0 0%
**≥ 0 and < 1 mm**	0 0%	0 0%	6 14%	2 4%	8 19%	2 4%	16 37%	1 2%
**≥ 1 and < 2 mm**	29 67%	6 11%	14 33%	3 5%	16 37%	3 5%	12 28%	4 7%
**≥ 2 mm**	14 33%	51 89%	21 49%	52 91%	13 30%	52 91%	12 28%	52 91%

Data is given as n (%)

Consequently, the difference between the patient’s bronchial diameter and the chosen Mallinckrodt DLT’s outer bronchial diameter also significantly differed between the methods (*P* < 0.001***) and sexes (*P* < 0.001***) (Fig. 2; Table 4). Current selection methods did not always meet the ≥ 1 mm criterion (difference < 1 mm: Slinger et al. [5]: 10%, Brodsky et al. [12]: 16%, Hannallah et al. [13]: 20%; Fig. 2, black line, Table 4) and occasionally selected tubes with an outer DLT’s bronchial diameter greater than the patient’s bronchial diameter in women (difference < 0 mm: Slinger at al. [5]: 5%, Brodsky et al. [12]: 14%, Hannallah et al. [13]: 7%; Fig. 2, red line, Table 4). In all methods, more than 89 to 91% of all men had a difference ≥ 2 mm.

## Discussion

This study demonstrates that current DLT selection methods fail to accurately predict bronchial diameters and overlook biological and manufacturer-specific variability. Women are at risk of bronchial damage from over-sized DLTs, and men from under-sized DLTs. To minimize this risk, manual measurement of the DLT’s outer diameter and 3D-reconstruction for bronchial diameter may be advisable. This is particularly relevant in high-risk patients like small women with predisposing factors such as chronic obstructive pulmonary disease, inflammatory lesions of the tracheobronchial tree, chronic use of steroids, or advanced age [34].

In clinical practice, the anaesthesiologist’s personal assessment is frequently the primary determinant in decision-making, often based on the patient’s sex and height [35]. Though, several methods have been proposed for systematically selecting left-sided DLTs for one-lung ventilation. These current methods rely on accurately predicting patients’ bronchial diameter from demographic or 2D-radiographic measurements [5, 12, 13]. Slinger et al. suggested the use of sex and body height for DLT selection [5]. Based on cadaver studies, Brodsky and colleagues used a conversion factor of 0.68 to estimate the patient’s bronchial diameter from the tracheal diameter to be used for 2D-radiographic measurements of the tracheal diameter [6, 12, 36]. In a later study, Brodsky et al. found ratios of 0.77 for men and 0.75 for women derived from 2D-CT scans [37]. Hannallah and others suggested measuring the bronchial diameter using 2D-CT [9, 13, 38]. However, our data show that biological variability in bronchial diameters makes these ratios inadequate for accurate DLT sizing, regardless of the method or X-ray magnification factor.

Current methods by Slinger et al., Brodsky et al., or Hannallah et al. resulted in selection tables, which select DLTs between 35 and 41 Fr in our study population and are currently in clinical use. Despite having the same goal of predicting bronchial diameter, these methods propose different DLT sizes for the same patients [7, 11].

For 79 to 89% of men, current selection methods suggest 41 Fr tubes. Other authors have suggested the use of 41 Fr DLTs for men before [39]. With this largest currently manufactured 41 Fr tube, 81 to 93% of men still have a ≥ 2 mm difference between the tube’s outer diameter and bronchial diameter, making size concerns less relevant for men.

For women, current methods achieve a < 1 mm difference in 19 to 44% of cases, and 5 to 14% receive DLTs exceeding their bronchial diameter, risking airway injuries. The prediction error ranges from 43 to 61%, with 2D-CT and chest X-ray methods showing prediction intervals between 5.9 mm and 8.3 mm. Adherence to these methods risks bronchial rupture in women by systematically choosing too-large DLTs [10, 40], a problem also noted in small Asian women [19, 41], leading to suggestions for modified criteria for this group [17]. Choosing over-sized DLTs may thus be a global problem in women.

For the ≥ 1 mm criterion to be applied, the patient’s bronchial diameter and the tube’s outer bronchial diameter need to be known [18]. Our study presumes that direct measurement of patients’ bronchial diameters by 3D reconstruction is the most accurate method to determine the bronchial diameter. It is superior to 2D-CT scans since the left main bronchus obliquely intersects the horizontal and coronary CT axis [13, 29–31]. Suggested methods measuring the bronchial diameter using 2D-CT scans have recently been shown to result in an accuracy of correctly selected DLTs of only 60% [38]. Given our data, the prediction error of current methods is too large to predict bronchial diameters accurately. Therefore, it may be beneficial to use the ≥ 1 mm criterion along with direct measurements of the patient’s bronchial diameter using 3D reconstruction and the tube’s outer bronchial diameter.

To add to the biological variability of patients’ bronchial diameters, it is well known that the outer bronchial diameter of the DLT varies considerably between the manufacturers [24, 26–28]. Since there is no industrial norm for the tubes’ outer bronchial diameter, a 35 Fr tube from the manufacturer, e.g., Rüsch, measures on average 9.95 mm, but a 39 Fr from Portex only 9.90 mm [24]. Current selection methods require manufacturer-specific tables, which is impractical. Any new manufacturer or DLT size would necessitate new tables with validation and error rate determination. Additionally, significant variability exists even within a single manufacturer’s tubes, as noted by other authors [20]. When selecting optimal tube sizes, one must consider the biological variability of patients’ airways and the technical variability of manufactured tubes and the manufacturer.

Selection methods based on the tracheal diameter in a chest X-ray are variable due to the distance of the trachea to the X-ray film. Greater distances result in more significant magnification and overestimation of the tracheal diameter [42]. An institutional-specific correction factor of about 10% is usually applied to address artificial magnification. Given the mean bronchial diameter in our study was 13.6 ± 2.1 mm, a 10% overestimation could eliminate the entire safety margin of ≥ 1 mm. Our study, intentionally working with this original approach, supports the > 1 mm selection criterion as a valid selection method. Furthermore, it is counter-intuitive that the Fr size on the packaging of the DLT does not refer to where the critical part of the DLT, namely the tube’s bronchial section, anatomically comes to rest. Other authors suggest margins between ≥ 0 mm and ≥ 2 mm [13, 18, 38]. Referencing current selection methods to a lower safety margin of < 1 mm would, however, lead to more women being exposed to a higher risk of bronchial damage. Since the anesthesiologist is recommended to be the ideal coordinator for preoperative assessment [43], they should be aware of the elevated risk this may pose to their patients.

3D reconstruction software is often included in routine radiological analysis, making it a minimal effort if a 2D-CT scan is already available. If 3D reconstruction seems tedious, it can be reserved for patients at higher risk of bronchial damage or rupture, such as women with chronic obstructive pulmonary disease, tracheobronchial inflammatory lesions, chronic steroid use, or advanced age [34].

## Conclusion

Three sources of variability affect DLT size selection: patients’ bronchial diameters and the between and within-manufacturer variability of tube diameters. Thus, current methods often fail to select accurate DLT sizes. Men can tolerate larger tube sizes in the left main bronchus, but women often receive too-large sizes. To minimize bronchial damage, measuring the tube’s outer diameter directly and using 3D reconstruction to measure bronchial diameter, especially in high-risk patients, seems advisable.

### Electronic supplementary material

Below is the link to the electronic supplementary material.

Supplementary Material 1

## Data Availability

Data is provided within the manuscript or the supplementary information files.

## Abbreviations

2D: two dimensional
3D three: dimensional
2D: CT two-dimensional computer chest tomography
DLT Double: Lumen Tube
Fr: French
SD: Standard Deviation
