# Supplementary material for: Common double-lumen tube selection methods overestimate adequate tube sizes in individual patients – a 3D reconstruction study
Source: BMC Anesthesiol. 2024 Jul 1;24:215. doi: 10.1186/s12871-024-02605-7 (PMC11218118; doi:10.1186/s12871-024-02605-7)
Supplement: Supplementary file 1 — Supplementary Material 1 [file 12871_2024_2605_MOESM1_ESM.pdf]

SUPPLEMENTARY DIGITAL CONTENT

SUPPLEMENTARY FIGURE CAPTIONS

Supplementary Figure S1. Measurement methods for (A) the tracheal diameter interclavicular in anterior-posterior chest X-ray, (B) the bronchial diameter 10 mm distal to the carina in a 2D coronary thorax CT imaging, (C) the 3D reconstruction of the airways and (D) the diameters of the left main bronchus in 3D reconstruction 10 mm distal of the carina.

Supplementary Figure S2. Visual illustration of the concepts of confidence and predictability in statistical analysis.

Supplementary Figure S3. Bland-Altman plots comparing left bronchial diameter measured by 3D reconstruction and 2D thorax CT (A) and tracheal diameter measured by 3D reconstruction and chest X-ray (B) (---) indicate the mean differences ± 2 standard deviations with their respective 95%-confidence intervals (···). Red lines show the negative bias for large mean values.

Supplementary Figure S4. Prediction of the tracheal diameter from 3D reconstruction depending on (A) demographic variables, (B) tracheal diameter in chest X-ray and (C) tracheal diameter in 2D thorax CT by linear models. Measured tracheal diameters are given as coloured dots depending on sex. Red lines show the tracheal diameter predictions and the 95%-prediction intervals as shaded areas. Vertical black line segments indicate the difference between measured and predicted tracheal diameters (individual prediction errors).

SUPPLEMENTARY FIGURES

Supplementary Figure S1

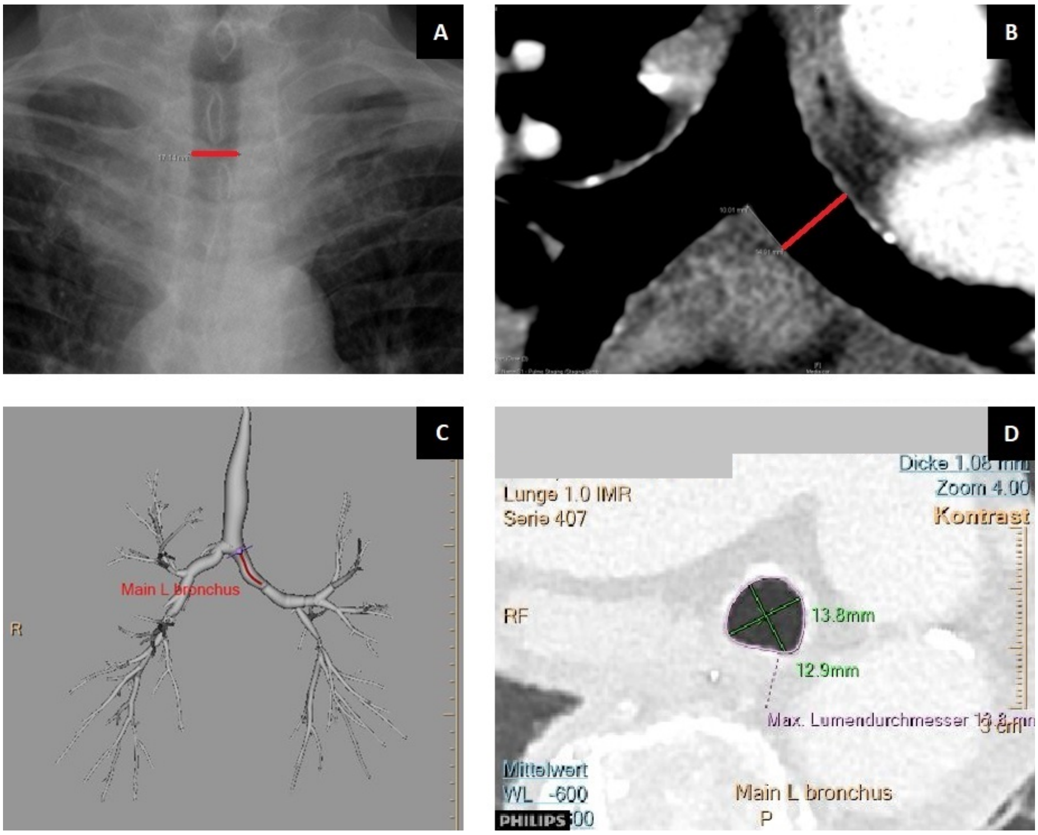

Supplementary Figure S2

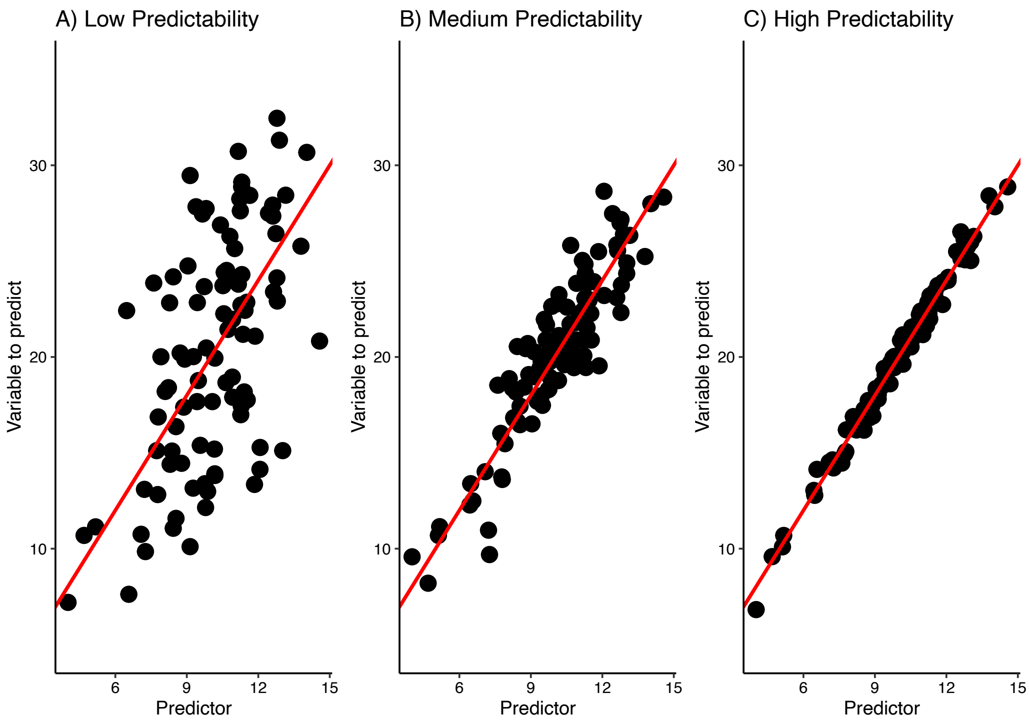

Supplementary Figure S3

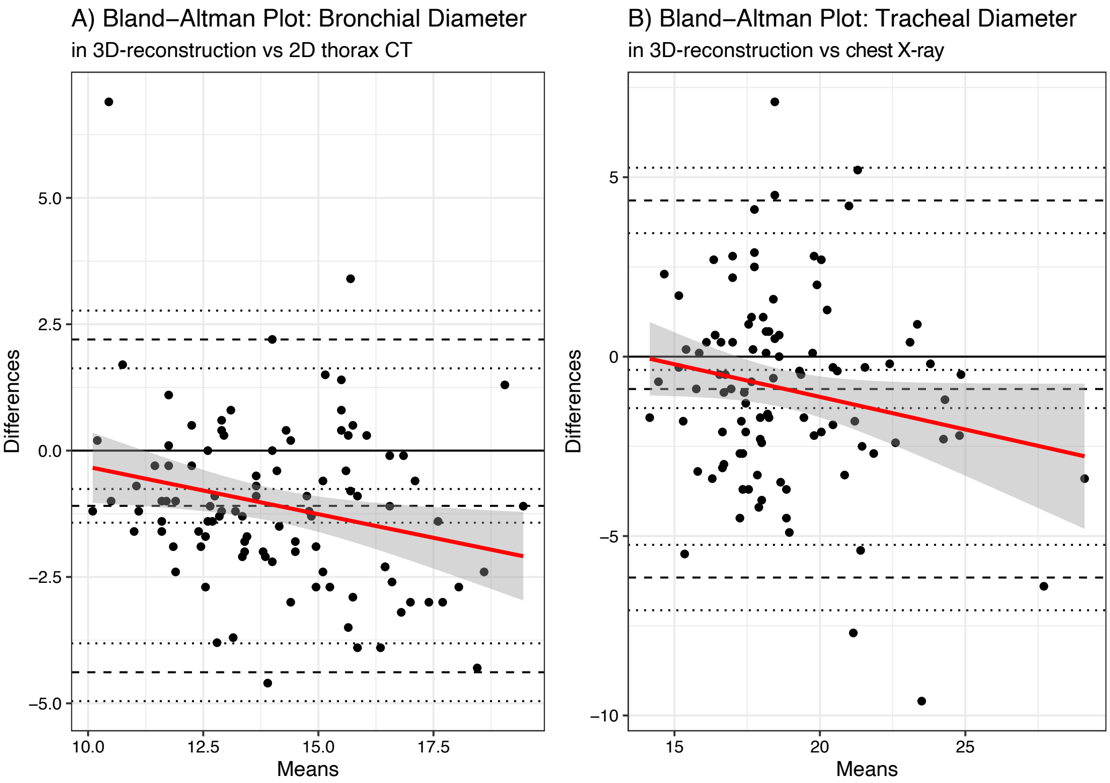

Supplementary Figure S4

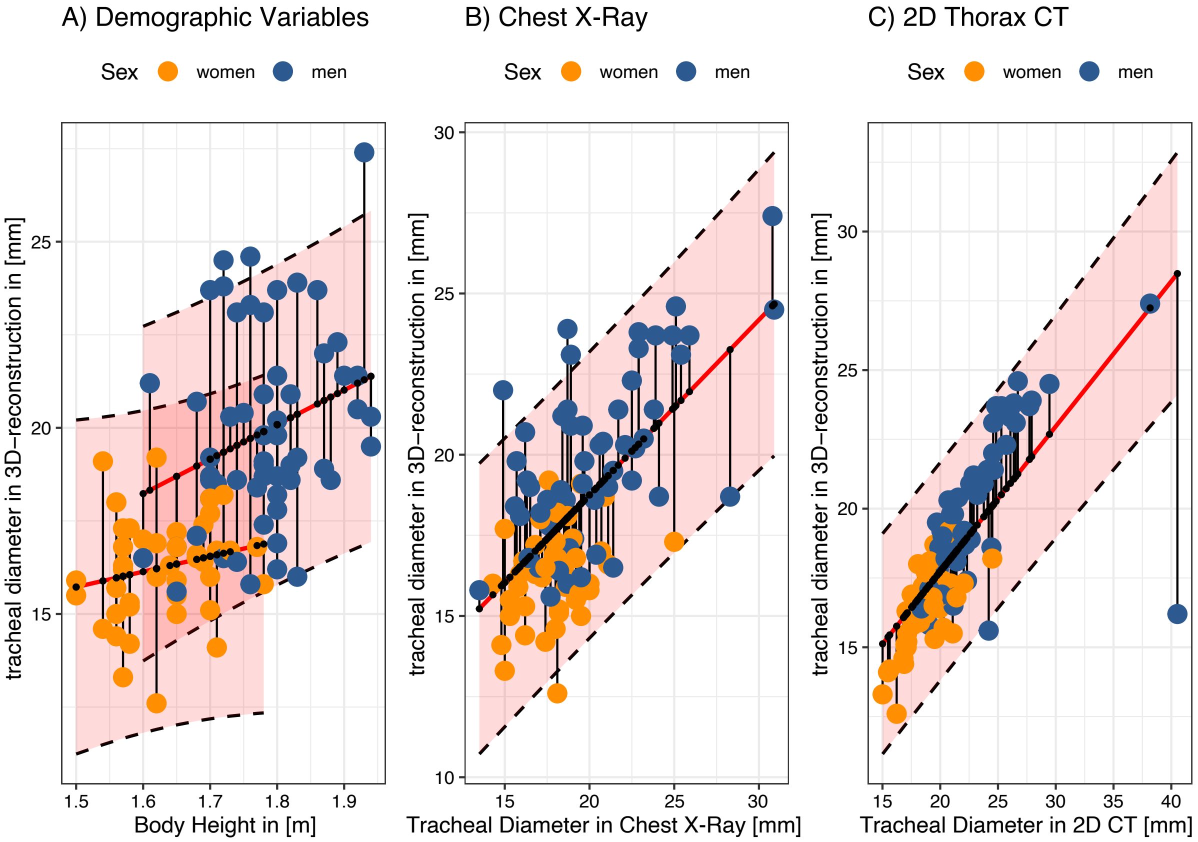

SUPPLEMENTARY TABLES

Supplementary Table S1. *Average outer diameter for the bronchial section of the double-lumen tube in [mm] for different manufacturers.*

Fr	Mallinckrodt[a]	Sheridan[b]	Portex[b]	Rüsch[b]	VIVASIGHT[b]	Well Lead[b]	EPSA[c]
32	7.40	n.a.	n.a.	n.a.	n.a.	n.a.	n.a.
35	9.60	9.80	9.25	9.95	9.70	9.65	9.83
37	10.10	9.95	9.35	10.05	9.80	10.45	10.45
39	10.50	10.00	9.90	11.00	10.05	10.55	10.92
41	10.80	10.70	10.55	11.40	10.65	10.80	11.22
[a]Data from [33], [b]Data from [25], [c]Measured diameters with calliper							

Supplementary Table S2. *Measured diameters from EPSA double-lumen tubes in [mm].*

Fr	*n*	Diameter
35	30	9.83 ± 0.18
37	40	10.41 ± 0.16
39	53	10.90 ± 0.12
41	18	11.23 ± 0.23
Data is given as mean ± SD.		

Supplementary Table S3. *Selected double-lumen tube sizes based on the ≥1 mm criterion applied to different double-lumen tube manufacturers.*

**Fr**	**Mallinckrodt**			**Sheridan[a]**			**Portex[a]**			**Rüsch[a]**			**VIVASIGHT[a]**			**Well Lead[a]**			**EPSA[a]**		
	**W**	**M**	**T**	**W**	**M**	**T**	**W**	**M**	**T**	**W**	**M**	**T**	**W**	**M**	**T**	**W**	**M**	**T**	**W**	**M**	**T**
**32**	5<br>12%	0<br>0%	**5**	7<br>16%	0<br>0%	**7**	3<br>7%	0<br>0%	**3**	10<br>23%	1<br>2%	**11**	5<br>12%	0<br>0%	**5**	5<br>12%	0<br>0%	**5**	8<br>19%	0<br>0%	**8**
**35**	5<br>12%	1<br>2%	**6**	3<br>7%	1<br>2%	**4**	1<br>2%	0<br>0%	**1**	0<br>0%	0<br>0%	**0**	2<br>5%	0<br>0%	**2**	11<br>26%	1<br>2%	**12**	8<br>19%	1<br>2%	**9**
**37**	6<br>14%	0<br>0%	**6**	0<br>0%	0<br>0%	**0**	4<br>9%	0<br>0%	**4**	13<br>30%	1<br>2%	**14**	3<br>7%	1<br>2%	**4**	1<br>2%	0<br>0%	**1**	7<br>16%	1<br>2%	**8**
**39**	5<br>12%	1<br>2%	**6**	10<br>23%	1<br>2%	**11**	9<br>21%	1<br>2%	**10**	6<br>14%	2<br>4%	**8**	10<br>23%	1<br>2%	**11**	4<br>9%	1<br>2%	**5**	3<br>7%	1<br>2%	**4**
**41**	22<br>51%	55<br>96%	**77**	23<br>53%	55<br>96%	**78**	26<br>60%	56<br>98%	**81**	14<br>33%	53<br>93%	**67**	23<br>53%	55<br>96%	**78**	22<br>51%	55<br>96%	**77**	17<br>40%	54<br>95%	**71**

[a]For Manufacturers without data for 32 Fr double-lumen tube diameters, a size <35 Fr is needed, 32 Fr is suggested to be the fitting size. W = women, M = men, T = total. Data is given as n (%).

Supplementary Table S4. *Prediction criteria of the three linear models for the tracheal diameter.*

**Model**	**R-squared**[a]	**Mean PE**[a]	**Median PE**[a]	**Max Span of 95%-Prediction Interval**
Demographic	0.37	2.3 mm	1.8 mm	9.1 mm
RTx	0.37	2.3 mm	1.8 mm	9.4 mm
2D-CTx	0.40	2.3 mm	1.4 mm	8.7 mm

Demographic: Sex and body height, RTx: tracheal diameter measured in chest X-ray, and 2D-CTx: tracheal diameter measured in 2D thorax CT scan, used as predictor variables.

PE: Prediction error; [a]: leave one out cross-validated.
